# Treatment of Complex Proximal Humeral Fractures in the Elderly with Reverse Shoulder Arthroplasty

**DOI:** 10.1111/os.12777

**Published:** 2020-10-04

**Authors:** Xu Tian, Ming Xiang, Guangyu Wang, Bo Zhang, Junyang Liu, Chao Pan, Lintao Liu, Jingming Dong

**Affiliations:** ^1^ Department of No.2 Upper Extremity Traumatology Tianjin Hospital Tianjin China; ^2^ Department of Upper Extremity Traumatology Sichuan Provincial Orthopaedic Hospital Chengdu China

**Keywords:** Arthroplasty, replacement, Elderly, Shoulder fractures

## Abstract

**Objective:**

To assess the short‐term efficacy of reverse shoulder arthroplasty in the treatment of complex proximal humeral fractures in the elderly.

**Methods:**

Forty‐three elderly patients treated operatively for complex proximal humeral fractures with reverse shoulder arthroplasty from July 2017 to January 2019 were retrospectively reviewed. To be specific, 12 males and 31 females were reviewed with an average age of 72.0 years (range, 66–78 years). All fractures were attributed to trauma and treated for 8.0 days on average (range, 6–11 days). As suggested from Neer classification, 21 cases (48.8%, 21/43) were three‐part fractures, and 22 (51.2%, 22/43) were four‐part fractures. To assess the postoperative efficacy, Visual Analog Scale (VAS), American Society of Shoulder and Elbow Surgery Shoulder Joint Score (ASES), Constant–Murley score and radiological examination were adopted. The Neer three‐part fracture group and the Neer four‐part fracture group were compared.

**Results:**

There was no significant difference in age, gender, operation time, and follow‐up period between Neer three‐part fracture group and Neer four‐part fracture group. All operations were successfully performed, and the average operation time was 120–170 min, with an average of 141.3 min. Besides, the mean blood loss was 407.0 mL (250–700 mL), and the average intraoperative blood transfusion reached 446.5 mL (400–800 mL). All patients received the follow‐up for 6 to 16 months, that is for 10.9 months on average. All patients were discharged in 7 days after operation, and no wound‐related complications were identified. In 8 weeks, the greater and lesser tuberosities of all patients healed completely. During the last follow‐up, no loosening or dislocation of prosthesis was detected, and the forward elevation of 133.0 (100°‐ 165°), the external rotation of 29.5° (20°–35°), the internal rotation of 46.7°(30°–60°), the VAS score of 0.8(0–3), the ASES score of 89.1(78.8–100.0) were achieved. Constant‐Murley score reached 88.7 (range, 70–98). There was no significant difference between Neer three‐part fracture group and Neer four‐part fracture group (*P* > 0.05). A 71‐year‐old patient developed the symptoms of axillary nerve injury after operation; he recovered completely at 6 weeks after the operation, which had not adversely affected the functional rehabilitation exercise or the stability of the prosthesis. At the follow‐up, no other complications (e.g., infection, acromial stress fracture, and scapular notching) were identified in all patients.

**Conclusion:**

The short‐term efficacy of one‐stage reverse shoulder arthroplasty to treat complex proximal humeral fractures in the elderly is satisfactory.

## Graphical abstract

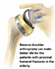

## Introduction

Proximal humeral fractures refer to the third most common osteoprotic fracture in the elderly. The incidence rate is only less than hip fracture and distal radius fracture^1^, ^2^. Though proximal humeral fractures can be largely treated in a conservative manner, operation is continuously adopted as the gold standard for the complex or severely displaced Neer three‐part and four‐part fractures^3^, ^4^. Existing surgical treatments of proximal humeral fractures primarily consist of internal fixation and arthroplasty^5^.

Complex proximal humeral fractures are capable of affecting the blood circulation of the humeral head. Even after internal fixation, the probability of humeral head necrosis can reach up to 35%^6^. Sometimes the comminuted fracture cannot be reduced and fixed, and the degeneration of rotator cuff may have occurred in the elderly patients before injury, so internal fixation may fail. In 1955, Neer initially treated complex proximal humeral fractures with hemi‐shoulder arthroplasty^7^. Subsequently, the method was extensively employed in clinical practice and has achieved a certain effect^8^, ^9^, ^10^. However, Zhuang reported numerous proximal humeral fracture with more significant tuberosity comminution or severe supraspinatus tendon injury^11^;on the whole, the effect of hemi‐shoulder arthroplasty is unsatisfactory. Kontakis *et al*.^12^ highlighted that for the proximal humeral fracture with greater tuberosity comminution or supraspinatus tendon injury, the major complication after hemi‐shoulder arthroplasty is poor function, especially shoulder elevation. Accordingly, surgeons had gradually highlighted the reverse shoulder arthroplasty, and such prosthesis was initially applied for the irreparable supraspinatus tendon injury^13^. At present, the most used reverse shoulder prosthesis was designed by French doctor Paul Grammont, and the concept refers to moving the rotatory center of the shoulder inward and downward. The force of deltoid muscle increases, so more deltoid muscle fibers can impact the activities of the shoulder. The deltoid muscle is extended, thereby enhancing the effect of deltoid muscle in shoulder activities, so the shoulder elevation is no longer dependent of the integrity of the rotate cuff. Sirveaux *et al*.^14^ initially reported the results of a large sample study on the reverse shoulder arthroplasty, and the elevation was averaged as 138° in patients after operation.

Since 2006, numerous reports have been made on the treatment of complex proximal humeral fractures with reverse shoulder arthroplasty, and satisfactory results have been achieved^15^, ^16^, ^17^, ^18^. However, rare reports have been made in China, especially the large sample retrospective study on the short‐term efficacy of the Grammont reverse shoulder arthroplasty for treating complex proximal humeral fractures.

Since July 2017, the reverse shoulder arthroplasty has been applied for the complex proximal humeral fractures in the elderly (aged 66–78 years). In the present study, the mentioned patients were retrospectively analyzed. The aim was to: (i) assess the short‐term effect of the reverse shoulder arthroplasty for the complex proximal humeral fractures in the elderly; (ii) discuss the operation precautions and complications in the reverse shoulder arthroplasty; and (iii) analyze the prevention of complications.

## Patients and Materials

### *Inclusion and Exclusion Criteria*

Inclusion criteria: (i) Neer three‐part or four‐part proximal humeral fractures; (ii) reverse shoulder arthroplasty; (iii) cases had complete clinical follow‐up data; (iv) retrospective series of case studies.

Exclusion criteria: (i) age < 65 years; (ii) pathological fracture of proximal humerus; (iii) axillary nerve injury or deltoid muscle injury before operation; (iv) incomplete follow‐up for less than 6 months.

### *Group Allocations*

According to Neer classification, two groups of patients were included in this study: three‐part group and four‐part group.

### *General Information*

From July 2017 to January 2019, 186 cases of proximal humeral fractures had undergone surgical operations in the Upper Extremity Department of Tianjin Orthopaedics Hospital, which specializes in orthopaedic diseases. Abiding by the inclusion and exclusion criteria, 43 cases were covered in this study. To be specific, 12 cases were male (27.9%, 12/43), and 31 were female (72.1%, 31/43), and these patients were aged 66–78 years, reaching 72.0 years on average. The mechanism was falling in 39 cases (90.7%, 39/43), and four cases experienced traffic injury (9.3%, 4/43); all of them had acute injury (less than 14 days). Thirty‐three cases were dominate side (76.7%, 33/43), 10 cases were non‐dominate side (23.3%, 10/43). The period from injury to operation was 6–11 days, 8 days on average. Routine radiographs covered anterior/posterior position, scapula Y position and axillary lateral position of the injury shoulder were taken. A computed tomographic scans help gain insights into the fracture. Magnetic resonance imaging is adopted to assess the degree of rotator cuff damage. According to Neer classification, there were 21 cases of three‐part fracture (48.8%, 21/43) and 22 cases of four‐part fracture (51.2%, 22/43), which included 23 cases of comminuted fracture (over three fragments) of greater tuberosity (53.5%, 23/43) and seven cases of Ellman III° injury of supraspinatus tendon (16.3%, 7/43), that is closed fractures without other combined injuries on the whole.

This study was approved by the ethics committee of the hospital and was subject to its supervision. Informed consent was acquired from all patients or their family members, and the present study complied with the provisions of the Declaration of Helsinki (as revised in Brazil in 2013).

### *Surgical Technique*

#### *Anesthesia*

General anesthesia was adopted, and the endotracheal tube should be taped to the contralateral side to avoid interference with the surgical field.

#### *Approach*

**Fig 1:**
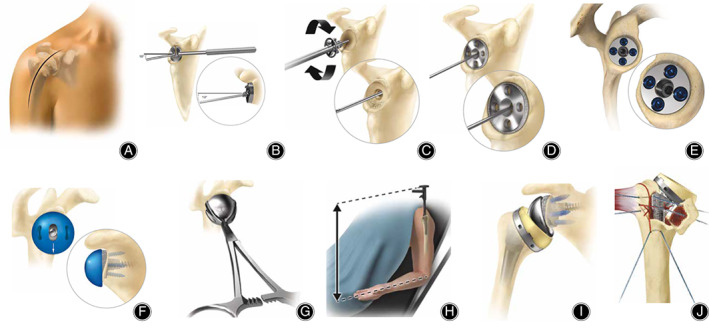
Operation diagram. (A) Utilize beach chair position and deltopectoral approach. (B) Insert a 3.2 mm Steinmann pin into the glenoid. (C). Ream the glenoid. (D) Due to the 10 degree inferior tilt of the Steinmann pin, the baseplate should also has a 10 degree inferior tilt. (E) Fix the basebase with 6.5 mm central screw and 4.75 mm peripheral screws. (F) Determine the amount and orientation of glenosphere offset. (G) Engage the glenosphere using the forceps. (H) Cement prosthesis was used in humeral side, the posterior tilt angle of prosthesis is all placed at 20°. (I) Assemble the humeral tray and bearing. (J) Tuberosity reconstruction with 5# Ethibond non‐absorbable sutures.

**Fig 2:**
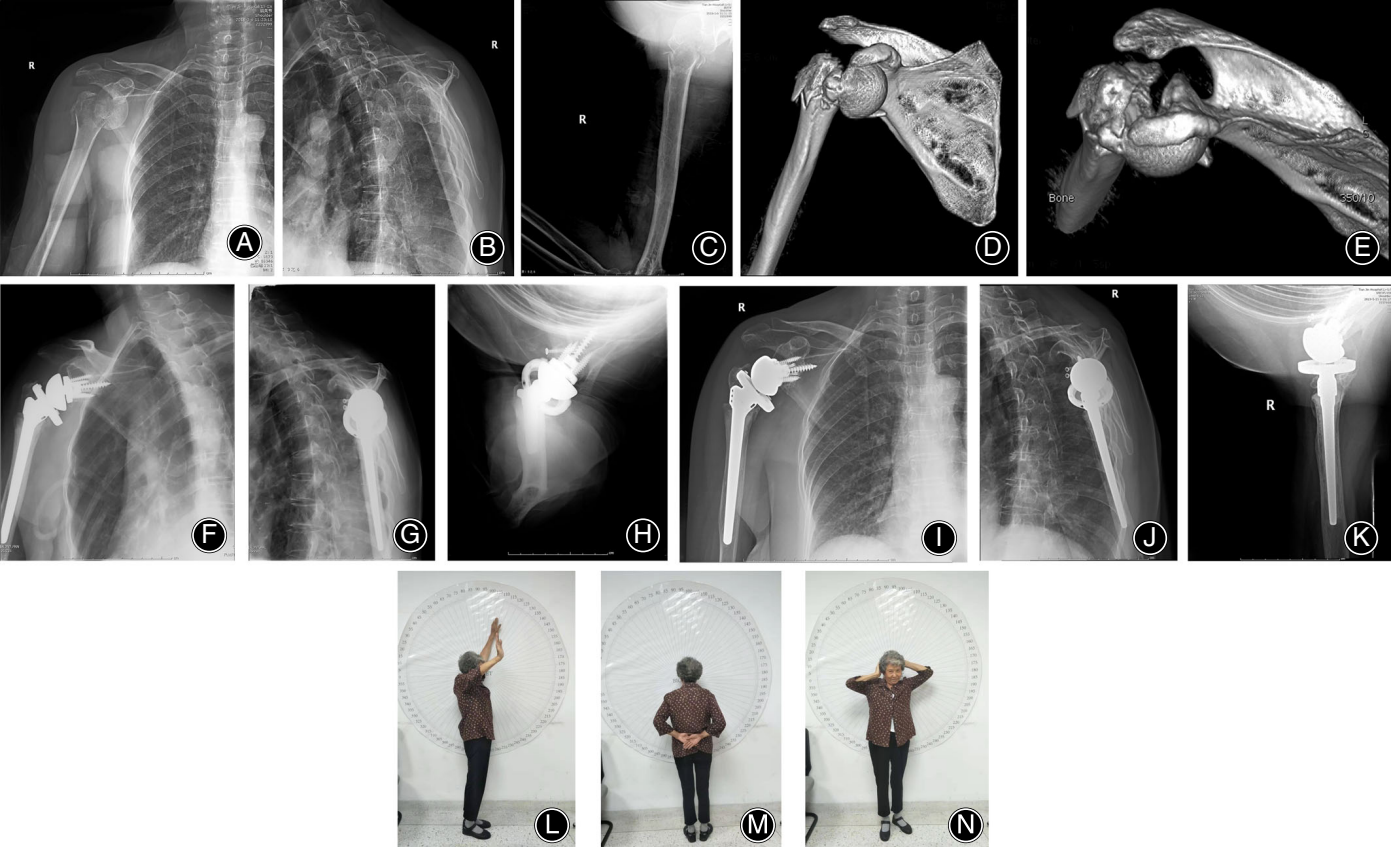
A 71 year‐old female patient with right proximal humeral comminuted fracture dislocation (Neer four part fracture dislocation) caused by a fall. (A–E). X‐rays and 3D‐CT showed comminuted fracture of proximal humerus with dislocation of glenohumeral joint. (F–H). The X‐rays in the AP view, Y view and axillary view indicated that the prosthesis position was satisfactory after the reverse shoulder arthroplasty. (I–K). At the end of 14 months follow‐up after operation, the X‐ray films of the AP view, Y view and axillary view showed satisfactory healing of the greater and lesser tuberosities. (L–N). At the end of 14 months follow‐up after operation, the forward elevation was 125°; external rotation was 35°; internal rotation was 50°. VAS score was 0°; The Neer score was 93; Constant‐Murley score was 83.

**Fig 3:**
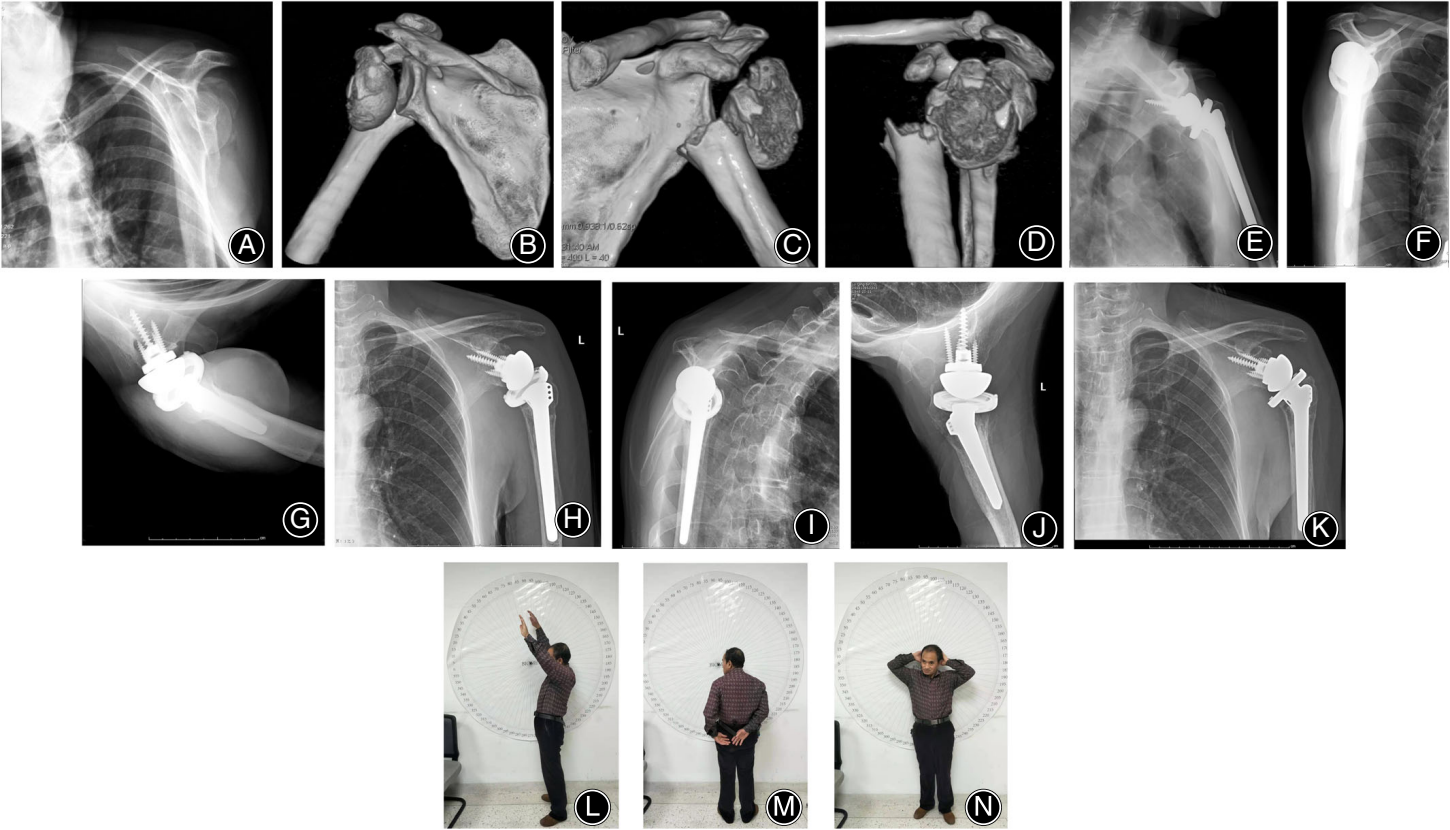
A 70 year‐old male patient with left proximal humeral comminuted fracture (Neer three part fracture) caused by a fall. (A–D). X‐rays and 3D‐CT showed comminuted fracture of proximal humerus with serious compress fracture of the greater tuberosity. (E–G). The X‐rays in the AP view, Y view and axillary view indicated that the prosthesis position was satisfactory after the reverse shoulder arthroplasty. (H–J). At the 5 months follow‐up after operation, the X‐ray films of the AP view, Y view and axillary view showed satisfactory healing of the greater and lesser tuberosities. (K). At the 7 months follow‐up after operation, the AP view indicated that the prosthesis position was satisfactory. (L‐N). At the 7 months follow‐up after operation, the forward elevation was 150°; external rotation was 30°; internal rotation was 40°. VAS score was 0°; The Neer score was 94; Constant‐Murley score was 96.

The patient was positioned in beach chair position, the arm and shoulder were prepped and draped free. During the operation, the deltopectoral approach (Fig. 1A) was employed, and the cephalic vein was routinely taken medially. The deltoid and pectoralis were exposed, and their insertions were protected. The long head tendon of biceps was identified after the release of the clavipectoral fascia. Besides, the greater and lesser tuberosities were recognized (Fig. 2). The interval between supraspinatus tendon and subscapular tendon was opened, the insertions of rotate cuff were tagged with 5# Ethibond non‐absorbable sutures. Subsequently, the greater and lesser tuberosities were pulled separately to expose the humeral head. After the humeral head was taken out, the glenoid was exposed. The long head tendon of biceps was cut off at the insertion of the supraglenoid tubercle. The supraspinatus tendon was removed routinely (Fig. 3).

#### *Glenoid Preparation*

The Hohmann shoulder retractors were employed to fully expose the glenoid, and then the labrum was resected. A retractor shoulder was placed at the inferior aspect of the glenoid to avoid injury of the axillary nerve. The glenoid guide handle was attached to the glenoid and a 3.2 mm Steinmann pin was inserted into the glenoid at the desired angle and position (Fig. 1B). Subsequently, the cannulated baseplate reamer was positioned over the top of the Steinmann pin. The glenoid was reamed to the desired level, and then the medial geometry of the glenoid baseplate was ensured to be completely reamed (Fig. 1C). It is noteworthy that the glenoid was adequately reamed to ensure the complete seating of the glenoid baseplate. As impacted by the 10° inferior tilt of the Steinmann pin, the baseplate should have a 10° inferior tilt (Fig. 1D), and then the basebase was fixed with 6.5 mm central screw and 4.75 mm peripheral screws (Fig. 1E). The appropriately sized glenosphere trial was selected and assembled to a trial taper adaptor. The amount and orientation of glenosphere offset were determined, and it was noted that a fully inferior tilt glenosphere created the optimal opportunity to minimize or eliminate scapular notching (Fig. 1F). Subsequently, the glenosphere was engaged with the forceps, and the glenosphere was implanted in the identical orientation as the trial (Fig. 1G).

#### *Humeral Preparation and Assembly*

Cement prosthesis was employed in humeral side. The height of prosthesis could be measured in accordance with the height of medial calcar or the standard of 5 cm above the pectoralis insertion; the posterior tilt angle of prosthesis was placed overall at 20° (Fig. 1H). The tightness of the prosthesis was tested, and then the humeral tray and bearing were assembled. The joint was reduced as assisted by the shoe horn, and the final range of motion was assessed (Fig. 1I). Tuberosities were reduced around the neck of the stem with 5# Ethibond non‐absorbable sutures (Fig. 1J). Cancellous bone graft was required between the greater and lesser tuberosities to facilitate healing. The long head tendon of biceps was tuned and sutured to the short tendon. Afterwards, drainage was placed before the wound was closed.

### *Postoperative Management*

Antibiotics were routinely used 1 day postoperatively, and the drainage was removed in 48 h postoperatively. The shoulder was placed in a shoulder immobilizer (shoulder abduction 20°, elbow flexion 90°, and forearm neutral position). Physical therapist could facilitate the initial shoulder pendulum exercise and the active movement of elbow, wrist, and fingers on the first day postoperatively. The passive function exercise of shoulder was performed as supervised by physical therapists in the 6 weeks postoperatively. At 6 weeks, active assisted motion of the shoulder was initiated.

### *Postoperative Assessments*

#### *Shoulder Joint Range of Motion*

All patients were examined for range of motion of shoulder joints at the last follow‐up. This included forward elevation, external rotation, and internal rotation.

#### *Visual Analogue Scale*

Visual analogue scale (VAS) was adopted to assess the pain. Out of a total score of 10, 0 represents no pain, 1–3 points for mild pain, 4–6 points for moderate pain, 7–9 points for severe pain, and 10 refers to unbearable pain.

#### *American Society of Shoulder and Elbow Surgery Shoulder Joint Score (ASES)*

The ASES score includes a patient self‐assessment section and a section completed by the doctor^19^. Patient section includes pain, stability, and daily activities, the doctor's section includes range of motion, strength, instability, and demonstration of specific physical signs.

#### *Constant–Murley* *Score*

The shoulder function was assessed by the Constant–Murley score. The score consists of four variables that are used to assess the function of the shoulder. The subjective variables include pain (15 points) and daily activities (20 points). The objective variables include active range of motion (40 points) and muscle strength (25 points). The overall score was 100 points.

#### *Imaging Examination*

Routine radiographs consisted of anterior/posterior position, scapula Y position, and axillary lateral position of the injured shoulder taken at follow‐up.

### *Statistical Methods*

All statistical analyses were performed with SPSS v22.0 (IBM, Armonk, NY, US). Continuous variables were presented as the mean ± standard deviation and were analyzed with two‐sided independent *t*‐test. Categorical variables were analyzed with Pearson chi‐squared test. *P* value < 0.05 was considered statistically significant.

## Results

### *General Results*

There was no significant difference in age, gender, operation time, and follow‐up period between three‐part group and four‐part group (Table 1). All the 43 patients were administrated with the Comprehensive reverse shoulder prosthesis (Comprehensive Reverse Shoulder System, Zimmer Biomet, USA), a second generation of Grammont prosthesis. The operation time was 120–170 min, with an average of 141.3 min. In the course of the operation, one of the patients was found to be complicated with Ideberg I A fracture of the scapula. Thus, the humeral head was pruned and fixed to the fracture site with screws; then, it was replaced routinely. All the 43 patients were discharged in 7 days after operation. No wound complications occurred at the time of discharge, and there was no delayed infection. All the sutures were taken out at 3 weeks postoperatively. All the patients received a follow‐up for 6–16 months, with an average of 10.9 months.

**TABLE 1:** Comparison of general informations of the 2 groups

Group	cases	Age (year)	Gender (male:female)	Operation time (day)	Follow‐up (month)
three‐part group	21	72.1 ± 4.3*	5:16	8.1 ± 1.5*	11.1 ± 2.7*
four‐part group	22	71.9 ± 3.7*	7:15	7.9 ± 1.7*	10.7 ± 2.8*
*P* value	‐	0.881	0.558	0.637	0.583

* , data were expressed as mean ± SD.

### *Shoulder Joint Range of Motion*

At the last follow up after the operation, the mean flexion elevation, the external rotation, and the internal rotation were 133.0° (range, 100°–165°), 29.5° (range, 20°–35°), and 46.7° (range, 30°–60°), respectively. There were no significant differences in shoulder joint range of motion between the two groups (*P* = 0.842, *P* = 0.989, *P* = 0.800) (Table 2).

**TABLE 2:** Comparison of shoulder function scores

Group	cases	forward elevation (°)	external rotation (°)	internal rotation (°)	VAS score	ASES score	Constant‐Murley score
three‐part group	21	133.6 ± 17.8	29.5 ± 5.2	46.4 ± 7.6	0.8 ± 0.9	89.5 ± 4.8	88.2 ± 8.3
four‐part group	22	132.5 ± 17.2	29.5 ± 4.9	47.1 ± 8.3	0.8 ± 1.0	88.8 ± 5.2	89.1 ± 6.5
*P* value	‐	0.842	0.989	0.800	0.900	0.618	0.723

Note: VAS, Visual Analogue Scale; ASES, American Society of Shoulder and Elbow Surgery Shoulder Joint Score.

### *Visual Analogue Scale (VAS) Score*

At the last follow‐up, all the 43 patients had no pain affecting sleep, 22 of which had slight pain, 19 could be relieved only by taking celecoxib occasionally, three had some pain relief, and none had their daily life impacted; eight of the patients reported that they had shoulder fatigue and discomfort after considerable activity, which was relieved after rest. VAS score of all patients reached 0–3, with 0.8 on average. There were no significant differences in the VAS score between the two groups (*P* = 0.900) (Table 2).

### *Fracture Healing and Prosthesis Position*

Routine radiographs consisted of anterior/posterior position, scapula Y position, and axillary lateral position of the injured shoulder taken at follow‐up. As revealed from the results of X‐ray examination, the greater and lesser tuberosities healed in 8 weeks. At the last follow‐up, none of the patients developed complications (e.g., prosthesis loosening, dislocation, and scapular notching).

### *American Society of Shoulder and Elbow Surgery Shoulder Joint Score (ASES)*

At the last follow‐up, all patients had stable shoulders. Five patients complained that they were inconvenient in daily life, whereas their self‐care function was not affected. ASES score ranged from 78.8 to 100.0, with 89.1 on average. There were no significant differences in the ASES score between the two groups (*P* = 0.618) (Table 2).

### *Constant–Murley* *Score*

At the last follow‐up, 43 cases of shoulder elevation was 100°–165°, reaching 133.0° on average; external rotation was 20°– 35°, with an average of 29.5°; internal rotation was 30°–60°, with 46.7° on average. Five patients complained about the insufficiency of active range of motion of shoulder, (e.g., elevation, internal rotation, and external rotation). The Constant–Murley score of all patients ranged from 70 to 98, with an average of 88.7. There were no significant differences in the Constant–Murley score between the two groups (*P* = 0.723) (Table 2).

### *Complications*

At the follow‐up, no other complications (e.g., infection, acromial stress fracture, and scapular notching) were detected in any of the patients. One 71‐year‐old patient developed symptoms of axillary nerve on the first day postoperatively, primarily manifesting as numbness of axillary nerve innervation area and weakening of deltoid contraction strength. After being administrated with neurotrophic drugs, the symptoms were mitigated at the reexamination at 6 weeks postoperatively, through which the functional rehabilitation exercise or the stability of the prosthesis was not affected.

## Discussion

### *Efficacy of the Reverse Shoulder Arthroplasty*

The initial indication of reverse shoulder arthroplasty is irreparable rotator cuff injury, whereas the indication has now been expanded (e.g., the complex proximal humeral comminuted fracture in the elderly, the revision of hemi‐shoulder arthroplasty, and tumor^20^). Compared with hemi‐shoulder arthroplasty, the most significant advantage of reverse shoulder arthroplasty is that it is not dependent on the supraspinatus muscle when lifting the shoulder. Its biomechanical principle refers to moving the rotation center of the shoulder medially and applying the deltoid to lift the affected limb completely. However, it is noteworthy that the rotation of the shoulder still depends on the rotator cuff. Accordingly, even if the reverse shoulder arthroplasty is done, the greater and lesser tuberosities should be restored and fixed firmly^21^. In the mentioned group, the greater and lesser tuberosities were fixed by multiple 5# Ethibond non‐absorbable sutures, and the tuberosities healed efficiently. At the last follow‐up, the shoulder could be actively rotated in a medial and lateral manner, which can satisfy the needs of daily life. Under the lateralized design, the glenohumeral articulation was placed close to the anatomic location of normal controls and better external rotation was imparted to patients.

Moreover, the shoulder prosthesis applied in this study is the second generation of Grammont reverse shoulder prosthesis, as characterized by the “neck free” design of the glenosphere, as an attempt to significantly reduce the probability of loosening. Increased lateralization design resulted in decreased impingement of the humeral cup with the inferior border of the scapula, and the clinical outcomes suggested that this may impact range of motion, with traditional designs achieving greater forward flexion and lateralized designs achieving greater external rotation. The glenosphere exhibits its own downward inclination of 10° to eliminate the potential for scapular notching maximally. The humeral side is significantly downward, increasing the range of shoulder activity^22^. At the last follow‐up, none of the 43 patients suffered from loosening or dislocation of the prosthesis. The forward elevation was 100°‐165°, with an average of 133.0°; external rotation reached 20°‐35°, with 29.5° on average; internal rotation was 30°‐60°, with an average of 46.7°, which could satisfy the needs of daily life. Thus, the results of this study suggested that lateralized glenosphere designs are associated with less humeral and scapular impingement and therefore associated with lower scapular notching rates. Moreover, lateralized glenosphere designs may also be a better solution for medially eroded glenoids since they move the joint line more laterally to more effectively restore its native position, potentially enhancing joint stability and postoperative internal and external rotation.

### *Operation Precautions*

The reverse shoulder arthroplasty refers to a type of non‐anatomical prosthesis essentially. Choi *et al*. reported that the clinical outcomes of reverse total shoulder arthroplasty (RTSA) were satisfactory with overall complication rates of 15.7%. An orthopaedic surgeon in the learning curve period for the operation of RTSA should be rigorous when selecting the patients and performing RTSA^23^. The following aspects should be stressed during the operation. (i) The height of the prosthesis at the humeral side and the posterior tilt angle. The stability of the prosthesis with the tension of deltoid should be maintained, and the elevation of the shoulder should be completed after the reverse shoulder arthroplasty. However, when the size and angle of the glenosphere are fixed, the tension of deltoid is determined by the height of the humeral stem. The height of prosthesis stem in fracture patients is difficult to judge for the separation of greater and lesser tuberosities from metaphysis. The following methods are commonly adopted to determine the height appropriately: (a) before the operation, X‐ray examinations of the opposite side are performed to make comparison; (b) during the operation, the height of the prosthesis stem is judged according to the length of the medial calcar attached to the humeral head fragment; (c) the point marked on the pectoralis majoris, which is generally 5–6 cm from the top of the prosthesis. The posterior tilt angle of the prosthesis stem should not be excessively large, which is appropriate at 20. (ii) The true glenoid is identified. Since the elderly patients with proximal humeral fracture may have degeneration of shoulder before injury (e.g., hyperosteogeny), the lower edge of the glenoid in such patients will often thicken, thereby covering up the real glenoid, making it easy to misguide the surgeon to the hyperplastic area of the “glenoid.” If the glenosphere is installed in this scenario, it can not be fixed firmly, thereby causing early failure. (iii) Fixed pattern of greater and lesser tuberosities. Though the elevation of shoulder joint is not dependent on the greater tuberosities after the reverse shoulder arthroplasty, the external rotation still requires the participation of the rotator cuff. Accordingly, the greater and lesser tuberosities should be anatomically reduced. The encircling fixations of the greater and lesser tuberosities were performed to the prosthesis stem, greater tuberosity to stem, lesser tuberosity to stem, greater and lesser tuberosities to metaphysis. On the whole, 5# Ethibond non‐absorbable sutures can be employed for fixation, whereas cancellous bone should be implanted between the tuberosities to facilitate early healing.

### *Prevention of Postoperative Complications*

The postoperative complications are primarily associated with the surgeon's familiarity with prosthesis and the operation details. Only by fully understanding the procedure can complications be reduced. Kempton *et al*.^24^ highlighted that the common complications after reverse shoulder arthroplasty involve hematoma, infection, dislocation, acromion stress fracture, scapula notching, and others, with the maximum incidence of 68%; however, the incidence of complications will be significantly down‐regulated when the number of cases is more than 40. Thus, the learning curve of reverse shoulder arthroplasty is longer. Moreover, with the development of prosthesis design, the incidence of complications will decrease. Though the complications did not appear in our group, there was a transient axillary nerve injury in a 71‐year‐old patient, which was considered to show associations with stretching during operation. Since most of the patients with proximal humeral fractures are aged, the soft tissue is relatively weak, so surgeons should be highly rigorous to reduce stretching to prevent axillary nerve injury.

Moreover, dislocation and stress fracture of acromion are primarily due to the wrong judgment of the tightness of prosthesis. Scapular notching refers to a special complication of the reverse shoulder arthroplasty, which has been reported in 31.7% of all cases. Furthermore, the extent of scapular notching progresses with the length of follow‐up and determines a low Constant score when associated with abnormal humeral images^25^. The mentioned finding is because when the shoulder adducts, the humeral tray collides with the lower edge of the scapular neck, which will cause the loosening and even displacement of the glenosphere^26^. Nyffeler *et al*.^27^ and Roche *et al*.^28^, ^29^, ^30^ considered that the good judgment of glenosphere offset and declination can lower the incidence of scapular notching. No case of scapular notching was identified in our group, probably because of two reasons. (i) Improvement of prosthesis: lateralized glenosphere designs are associated with less humeral and scapular impingement and therefore are associated with lower scapular notching rates. Moreover, the glenosphere had a 10° downward tilt, capable of lowering the probability of scapular notching. (ii) The postoperative complications also display relationships to the surgeon's familiarity with prosthesis and the operation details. Only by fully understanding the procedure, the incidence of complications could be reduced. (iii) The follow‐up time of this study is relatively short, and perhaps after a longer follow‐up, scapular notching will be found.

### *Limitations*

This study has the following limitation: (i) the follow‐up time of this study is short, the current clinical efficacy is only limited to the early stage, and the long‐term efficacy requires subsequent follow‐up; (ii) though the results of this study are satisfactory, it is not a multicenter study, so it lacks the benefits of a multicenter, large‐ample clinical study; (iii) this study is only retrospective, and a prospective randomized clinical control study is required to enhance persuasiveness.

On the whole, one‐stage treatment of complex proximal humeral fractures in the elderly with reverse shoulder arthroplasty exhibits a satisfactory early efficacy; however, there is still a long learning process before putting the technique into practice. Thus, only by gaining a full insight into the principles and operation steps of the prosthesis and by recruiting experienced upper extremity surgeons can we achieve satisfactory clinical efficacy.

## References

[1] Yang GY , Xiang M , Chen H , Hu XC . Short‐term clinical outcome of proximal humeral fractures using Multiloc proximal humeral nail. Chin J Orthop, 2016, 36: 103–112.

[2] Passaretti D , Candela V , Sessa P , Gumina S . Epidemiology of proximal humeral fractures: a detailed survey of 711 patients in a metropolitan area. J Shoulder Elbow Surg, 2017, 26: 2117–2124. 28735839 10.1016/j.jse.2017.05.029

[3] Neer CS 2nd . Displaced proximal humeral fractures. II. Treatment of three‐part and four‐part displacement. J Bone Joint Surg Am, 1970, 52: 1090–1103. 5455340

[4] Rangan A , Handoll H , Brealey S , *et al* Surgical vs nonsurgical treatment of adults with displaced fractures of the proximal humerus: the PROFHER randomized clinical trial. JAMA, 2015, 313: 1037–1047. 25756440 10.1001/jama.2015.1629

[5] Xiang M , Hu XC , Jiang CY . Pay attention to the whole concept and improve the diagnosis and treatment of proximal humeral fracture. Chin J Orthop, 2017, 37: 1313–1317.

[6] Yüksel HY , Yılmaz S , Akşahin E , *et al* The results of nonoperative treatment for three‐ and four‐part fractures of the proximal humerus in low⁃demand patients. J Orthop Trauma, 2011, 25: 588–595. 21673601 10.1097/BOT.0b013e318210ea56

[7] Neer CS II . Articular repIacement for the humeral head. J Bone Joint Surg Am, 1955, 37: 215–228. 14367414

[8] Ferrel JR , Trinh TQ , Fischer RA . Reverse total shoulder arthroplasty versus hemiarthroplasty for proximal humeral fractures: a systematic review. J Orthop Trauma, 2015, 29: 60–68. 25186842 10.1097/BOT.0000000000000224

[9] Formaini NT , Everding NG , Levy JC , Rosas S . Tuberosity healing after reverse shoulder arthroplasty for acute proximal humerus fractures: the "black and tan" technique. J Shoulder Elbow Surg, 2015, 24: e299–e306. 26141197 10.1016/j.jse.2015.04.014

[10] Boileau P , Krishnan SG , Tinsi L , Walch G , Coste JS , Molé D . Tuberosity malposition and migration: reasons for poor outcomes after hemiarthroplasty for displaced fractures of the proximal humerus. J Shoulder Elbow Surg, 2002, 11: 401–412. 12378157 10.1067/mse.2002.124527

[11] Zhuang CY , Chen Z , Song YY , *et al* The incidence of rotator cuff tear in proximal humeral fractures and its correlation with fracture type and age distribution. Chin J Orthop, 2017, 37: 1356–1360.

[12] Kontakis G , Koutras C , Tosounidis T , Giannoudis P . Early management of proximal humeral fractures with hemiarthroplasty: a systematic review. J Bone Joint Surg Br, 2008, 90: 1407–1413. 18978256 10.1302/0301-620X.90B11.21070

[13] Lu B , Scarlat MM . Reverse shoulder arthroplasty: current trend in elective surgery. Chin J Orthop, 2018, 38: 627–634.

[14] Sirveaux F , Favard L , Oudet D , Huquet D , Walch G , Molé D . Grammont inverted total shoulder arthroplasIy in the treatment of glenohumeral osteoarthritis with massive rupture of the cuff. Results of a multicentre study of 80 shoulders. J Bone Joint Surg Br, 2004, 86: 388–395. 15125127 10.1302/0301-620x.86b3.14024

[15] Cazeneuve JF , Cristofari DJ . Grammont reversed prosthesis for acute complex fracture of the proximal humerus in an elderly population with 5 to 12 years follow‐up. Rev Chir Orthop Reparatrice Appar Mot, 2006, 92: 543–548. 17088750 10.1016/s0035-1040(06)75911-6

[16] Gallinet D , Clappaz P , Garbuio P , Tropet Y , Obert L . Three or four parts complex proximal humerus fractures: hemiarthroplasty versus reverse prosthesis: a comparative study of 40 cases. Orthop Traumatol Surg Res, 2009, 95: 48–55. 19251237 10.1016/j.otsr.2008.09.002

[17] Grubhofer F , Wieser K , Meyer DC , Catanzaro S , Schürholz K , Gerber C . Reverse total shoulder arthroplasty for failed open reduction and internal fixation of fractures of the proximal humerus. J Shoulder Elbow Surg, 2017, 26: 92–100. 27521139 10.1016/j.jse.2016.05.020

[18] Bufquin T , Hersan A , Hubert L , Massin P . Reverse shoulder arthroplasty for the treatment of three‐ and four‐part fractures of the proximal humerus in the elderly: a prospective review of 43 cases with a short‐term follow‐up. J Bone Joint Surg Br, 2007, 89: 516–520. 17463122 10.1302/0301-620X.89B4.18435

[19] Richards RR , An KN , Bigliani LU , *et al* A standardized method for the assessment of shoulder function. J Shoulder Elbow Surg, 1994, 3: 347–352. 22958838 10.1016/S1058-2746(09)80019-0

[20] Smith CD , Guyver P , Bunker TD . Indications for reverse shoulder replacement: a systematic review. J Bone Joint Surg Br, 2012, 94: 577–583. 22529074 10.1302/0301-620X.94B5.27596

[21] Longo UG , Petrillo S , Berton A , Denaro V . Reverse total shoulder arthroplasty for the management of fractures of the proximal humerus: a systematic review. Musculoskelet Surg, 2016, 100: 83–91. 27316439 10.1007/s12306-016-0409-0

[22] Routman HD , Flurin PH , Wright TW , Zuckerman JD , Hamilton MA , Roche CP . Reverse shoulder arthroplasty prosthesis design classification system. Bull Hosp Jt Dis, 2013, 73: S5–S14. 26631189

[23] Choi S , Bae JH , Kwon YS , Kang H . Clinical outcomes and complications of cementless reverse total shoulder arthroplasty during the early learning curve period. J Orthop Surg Res, 2019, 14: 53. 30777107 10.1186/s13018-019-1077-1 PMC6380013

[24] Kempton LB , Ankerson E , Wiater JM . A complication⁃based learning curve from 200 reverse shoulder arthroplasties. Clin Orthop Relat Res, 2011, 469: 2496–2504. 21328021 10.1007/s11999-011-1811-4 PMC3148393

[25] Cazeneuve JF , Cristofari DJ . Grammont reversed prosthesis for acute complex fracture of the proximal humerus in an elderly population with 5–12years follow‐up. Orthop Traumatol Surg Res. 2014, 100: 93–97. 24456760 10.1016/j.otsr.2013.12.005

[26] Mollon B , Mahure SA , Roche CP , Zuckerman JD . Impact of scapular notching on clinical outcomes after reverse total shoulder arthroplasty: an analysis of 476 shoulders. J Shoulder Elbow Surg, 2017, 26: 1253–1261. 28111179 10.1016/j.jse.2016.11.043

[27] Nyffeler RW , Werner CM , Gerber C . Biomechanical relevance of glenoid component positioning in the reverse Delta III total shoulder prosthesis. J Shoulder Elbow Surg, 2005, 14: 524–528. 16194746 10.1016/j.jse.2004.09.010

[28] Roche C , Flurin PH , Wright T , Crosby LA , Mauldin M , Zuckerman JD . An evaluation of the relationships between reverse shoulder design parameters and range of motion, impingement, and stability. J Shoulder Elbow Surg, 2009, 18: 734–741. 19250845 10.1016/j.jse.2008.12.008

[29] Roche CP , Marczuk Y , Wright TW . Scapular notching and osteophyte formation after reverse shoulder replacement: radiological analysis of implant position in male and female patients. Bone Joint J, et al., 2013, 95: 530–535. 23539706 10.1302/0301-620X.95B4.30442

[30] Roche CP , Marczuk Y , Wright TW , *et al* Scapular notching in reverse shoulder arthroplasty: validation of a computer impingement model. Bull Hosp Jt Dis, 2013, 71: 278–283. 24344620

